# Tissue engineering in reconstructive urology—The current status and critical insights to set future directions-critical review

**DOI:** 10.3389/fbioe.2022.1040987

**Published:** 2023-03-01

**Authors:** Karolina Ławkowska, Clemens Rosenbaum, Piotr Petrasz, Luis Kluth, Krzysztof Koper, Tomasz Drewa, Marta Pokrywczynska, Jan Adamowicz, the Trauma and Reconstructive Urology Working Party of the European Association of Urology Young Academic Urologists

**Affiliations:** ^1^ Department of Regenerative Medicine, Collegium Medicum, Nicolaus Copernicus University, Bydgoszcz, Poland; ^2^ Department of Urology Asklepios Klinik Barmbek Germany, Urologist in Hamburg, Hamburg, Germany; ^3^ Department of Urology Voivodeship Hospital Gorzów Wielkopolski, Gorzów Wielkopolski, Poland; ^4^ Department of Clinical Oncology and Nursing, Collegium Medicum, Nicolaus Copernicus University, Curie-Skłodowskiej 9, Bydgoszcz, Poland; ^5^ Department of Urology, University Medical Center Frankfurt, Frankfurt am Main, Germany

**Keywords:** tissue engineering, reconstructive urology, regenerative medicine, urology, biomaterials

## Abstract

Advanced techniques of reconstructive urology are gradually reaching their limits in terms of their ability to restore urinary tract function and patients’ quality of life. A tissue engineering-based approach to urinary tract reconstruction, utilizing cells and biomaterials, offers an opportunity to overcome current limitations. Although tissue engineering studies have been heralding the imminent introduction of this method into clinics for over a decade, tissue engineering is only marginally applied. In this review, we discuss the role of tissue engineering in reconstructive urology and try to answer the question of why such a promising technology has not proven its clinical usability so far.

## 1 Introduction

Tissue engineering has evoked hopes over the last few decades for new therapies aimed at replacing an injured or resected urethra, urinary bladder, or ureter. To offer this possibility, different biomaterials combined with cells were applied to create an artificial wall of the urinary tract, restoring function (Adamowicz et al., 2013). Advanced techniques of reconstructive urology supported by modern surgical tools are reaching their limits in terms of their ability to restore urinary tract function and a patient’s quality of life. Tissue engineering has been considered the ideal strategy to push reconstructive urology to the next level, where urologists would utilize cell-seeded biomaterials and stem cells in daily practice (Adamowicz et al., 2019a). The approach intended to create urological grafts, i.e., whole substitutes or tissues that can be implanted, regenerated, or permanently replace the urethra, urinary bladder, or ureter. Despite plenty of valuable research data revealing the biology of stem cells, the behavior of implanted adult stem cells, and the remodeling of biomaterial grafts within urinary tracts, tissue engineering is nowadays marginally influencing urological management. The tissue engineering-oriented sessions, so numerous in recent years, gradually disappeared from the scientific programs at essential urological meetings. This may be interpreted as a sign of skepticism about the relevance of tissue engineering for urological therapy. On the other hand, researchers responsible for the development of tissue engineering led to urologists’ awakening through the publication of results suggestive of groundbreaking outcomes. From the clinician’s perspective, the simplification of research models and the dominating positive interpretation of results made from tissue engineering achievements in urology produce few valuable reports.

This review aims to summarize and critically evaluate the role of tissue engineering in reconstructive urology and to provide informative data presenting the current status rather than focusing on remaining problems or glorifying achievements.

## 2 Current challenges in reconstructive surgery–Urological surgeon’s perspective

The current challenges in reconstructive urology consist of three major points. The first and most crucial challenge is to achieve the best oncologic results possible. The second challenge is to achieve the best functional results. The third driver in reconstructive surgery should be the goal of lowering morbidity at the explantation site.

New reconstructive material must fulfill these requirements. Due to the currently used reconstructive materials, it is no wonder that tissue-engineered materials or materials that do not need to be harvested, for example, amniotic membranes, have been investigated throughout the last century (Kaleli and Ansell, 1984). The primary driver of these efforts is the limited functional results and the side effects of tissue harvesting nowadays. Autologous tissues are used, i.e., intestinal segments for upper tract and bladder reconstruction, and skin or oral mucosa for urethral reconstruction (Xiong et al., 2020).

In bladder reconstruction, intestinal segments guarantee excellent oncological results. Recurrence-free survival in patients treated with radical cystectomy for bladder cancer ranges between 60% and 68% at five-year follow-up. Of those, local recurrences account for 30%–54% (Mari et al., 2018). Pathologic stage, lymph node invasion, the extent of lymphadenectomy, multifocality, and prostatic involvement were found to be independent predictors of pelvic recurrence, whereas the type of urinary diversion was not (Umbreit et al., 2010). In general, recurrence in the intestinal segment used to create a neobladder or ileal conduit is infrequent. However, the price for reasonable oncologic results is high. Radical cystectomies with urinary diversion come with an early complication rate of almost 100%, a 25% likelihood of readmission to the hospital, and a 5% risk of perioperative death (Vetterlein et al., 2019). Most of these side effects are related to resecting the intestinal segment. Gastrointestinal-related complications represent a significant portion of short- and long-term complications. Within the first 30 days postoperatively, almost 20% develop gastrointestinal problems. Further on, metabolic complications related to the use of the intestinal segment remain a long-term problem. Metabolic acidosis and chronic renal failure lower the life expectancy and quality of life of patients. Summed up, radical cystectomy and urinary diversion represent surgery-related high-risk complications. Almost all patients experience postoperative complications, and most of them can be explained as explantation site-related (Umbreit et al., 2010). Further functional results are also unsatisfactory. In the long term, complications that need to be operated on, such as stenosis of the ileal conduit or the ileal ureter anastomosis, are described in 12%–24% of cases (Lee et al., 2003) (Hautmann et al., 2011). This underlines the need for novel materials to be used for urinary diversion (Kloskowski et al., 2015a).

In urethral reconstruction, the oncologic outcome does not play a role, whereas functional results are of significant interest. Functional results depend on different stricture-related factors, such as bulbar stricture location or shorter strictures. If both facts come together, no substitution material should be used (Morey et al., 2014). Excision and primary anastomosis of the urethra are indicated and guarantee excellent results (Chapple et al., 2014). For penile or longer and more complex strictures, substitution for urethroplasty is indicated. Nowadays, buccal mucosa graft is the most commonly used substitution material. This substitution material has ruled out others like penile skin due to higher success rates. Still, success rates of buccal mucosa graft urethroplasties are lower than those of urethroplasties by excision and primary anastomosis. Success rates of buccal mucosa graft urethroplasties range between 70% and 87% (Vetterlein et al., 2018). When taking into consideration that these results include complex and lengthy strictures, the results can be seen as very good. Nevertheless, the side effects of harvest site grafting are not negligible. Efforts have been made to improve the management of the harvest site (Soave et al., 2018). However, oral complaints are common, especially in patients who require longer grafts due to longer strictures (Rosenbaum et al., 2016). Oral complaints consist of pain, bleeding, swelling, numbness, alteration of salivation and taste, and also impairment of mouth opening, smiling, whistling, diet, and speech. These complaints can reduce the quality of life for patients. Therefore, alternative substitution material has been sought and is currently being tested in clinical trials. A tissue-engineered oral mucosal graft (MukoCell^®^) has been used for the typical indication of buccal mucosa grafts and showed comparable success rates of 84% (Barbagli et al., 2018). Even though the application of this tissue-engineered substitution material is described as being more complicated than the application of buccal mucosa grafts, functional results are very encouraging.

## 3 Why can we offer so little to our patients?

**FIGURE 1:**
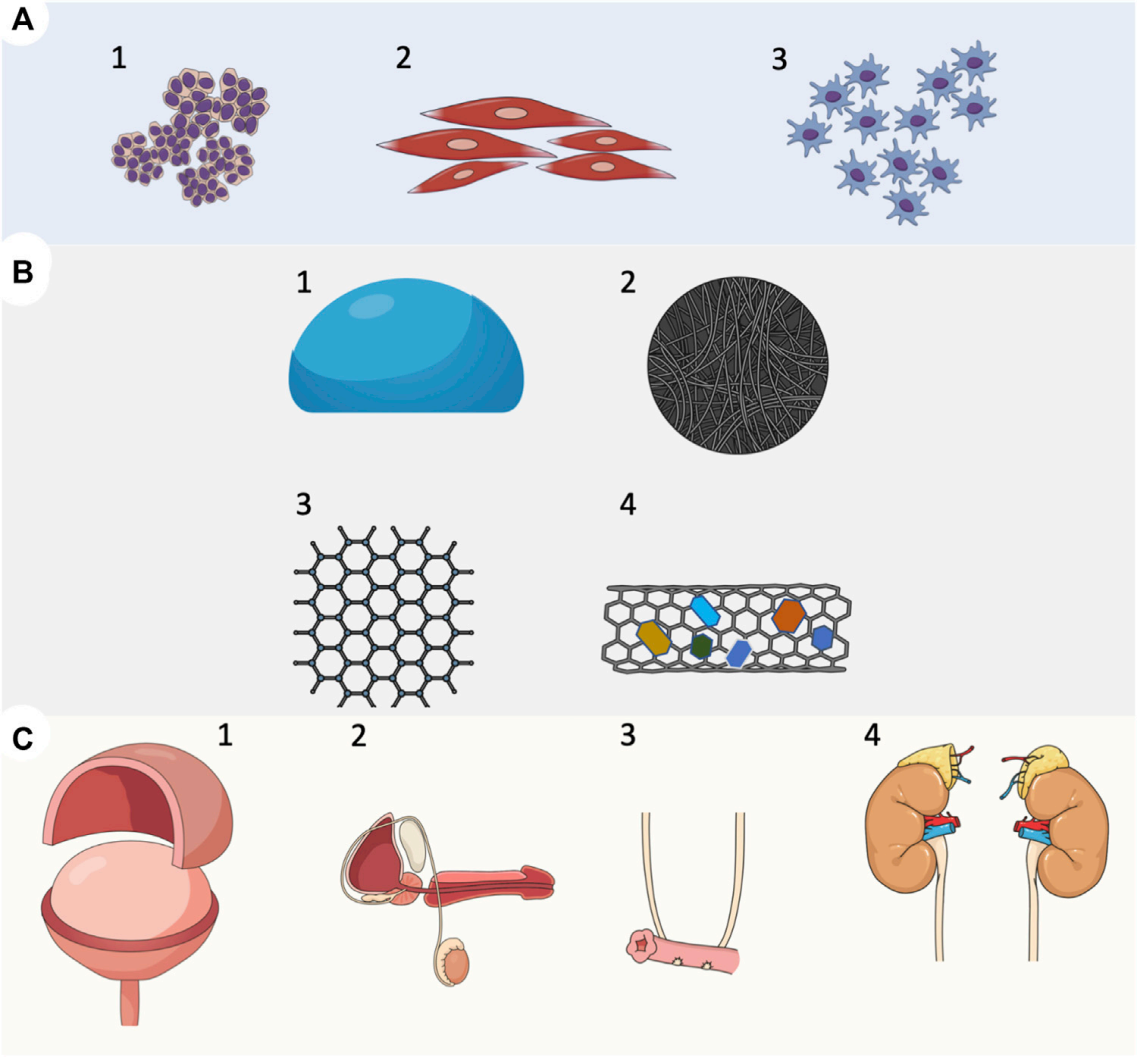
Overview of tissue engineering strategies in reconstructive urology. **(A)** Cell types used for the development of implantable grafts. (1) Urothelial cells; progenitors of urothelial cells derived from urine. (2) Smooth muscle cells derived from the bladder detrusor; smooth muscle cells progenitors derived from mesenchymal cells. (3) Mesenchymal cells derived from bone marrow or adipose tissue, **(B)** Biomaterials used in tissue-engineered urinary tract reconstruction. (1) Natural biomaterials, et cetera, amniotic membrane, small intestinal submucosa SIS (2) Decellularized scaffolds, et cetera. Acellular matrix of the bladder (BAM) (3) Polymer biomaterial, et cetera, collagen, gelatin, alginate, cellulose, and chitin (4) Scaffolds with incorporated bioactive components, et cetera, growth factors, smart biomaterials, **(C)** A tissue-engineered approach to experimental urinary tract reconstruction. (1) patch grafts aimed to replace urinary bladder wall in partial cystectomy model (2) patch grafts or tubular grafts for whole full-thickness urethral reconstruction (3) artificial urinary conduits (4) patch grafts or tubular grafts for ureteral reconstruction.

**FIGURE 2:**
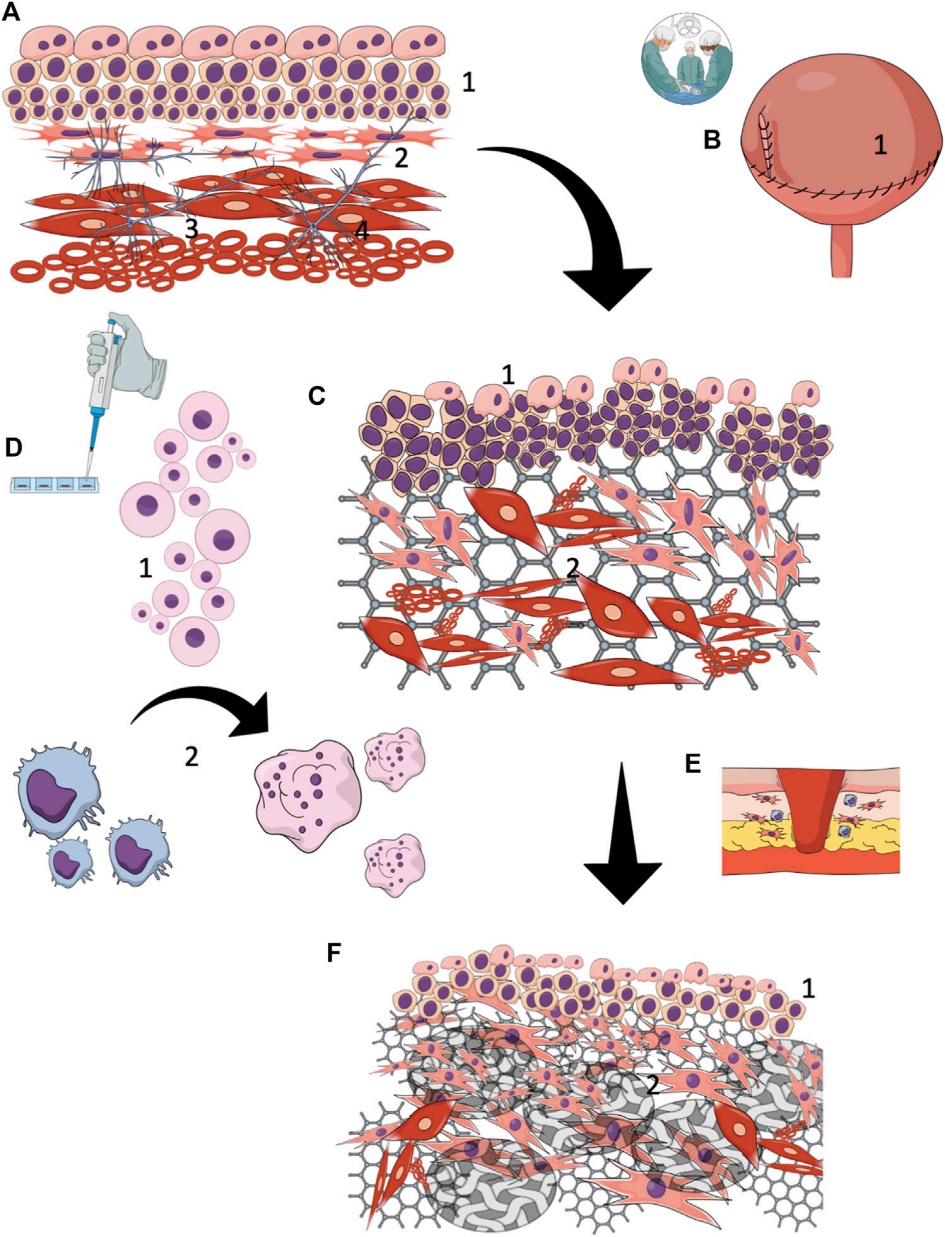
Tissue-engineered reconstruction of the urinary tract wall **(A)** A normal urinary tract wall, composed of a stratified urothelial layer (1) with interstitial cells beneath forming a syncytial-like layer (2) layered arrangement of the detrusor muscle layer (3) complex interstitial neuronal network (4), **(B)** Urinary tract wall reconstruction with cell-seeded grafts (1), **(C)** Early (< 3 months) regeneration results showed partial restoration of proper structure. All layers exhibited extensive structural and histological disturbances. Hypertrophic urothelium (1) with an incomplete external layer. No one of the studies focused on the regeneration of regulatory intestinal cells or the neuronal network. It is likely, however, that regeneration of these components does not occur. Extensive progressive fibrotic reaction due to ongoing inflammation and ischemia within the graft. Smooth muscle layer regrowth is irregularly arranged and gradually loses its native spatial configuration (2), **(D)** Seeded cells (1) are predominantly phagocytized (2) shortly after reconstruction by activated macrophages. The surveillance time of cells seeded on the biomaterial graft after implantation is not certain, but it is estimated at several weeks. In light of this data, seeded cells provide a temporary boost for limited natural regeneration mechanisms by supplying the environment with bioactive components, **(E)** With the persistence of inflammation, the tissue remodeling with the reconstructed urinary tract wall is oriented toward fibrosis , **(F)** The final reconstruction outcomes are not satisfying. Proper restoration of the urothelial layer is due to the high intrinsic regeneration potential of the epithelium. Nevertheless, it is unknown whether the complex regulatory function of urothelium is preserved. The biomaterial underwent efficient degradation and was replaced by collagen-rich scar tissue (2), covering most of the reconstructed area.

Whenever a new concept of medical therapy becomes available, it needs to be validated in clinical trials. Despite research efforts, tissue engineering is struggling to introduce reliable therapeutic options to clinics. Currently, only in the field of urethral reconstructive surgery have clinical trials involving tissue engineering shown relevant results, justifying the continuation of research (Kanematsu, 2018). Despite the media attention, research, and community interest, tissue engineering therapies did not reach the mainstream application. They are limited by their inability to effectively recapitulate the complex cellular, structural, and mechanical environment of native tissues when transitioning from *in vitro* to *in vivo* applications (Figures 1,2) (O’Donnell et al., 2019).

### 3.1 Questionable effectiveness in clinical trials

#### 3.1.1 Urinary bladder

The landmark study of Atala et al. (2006) aimed to augment the bladder in spina bifida patients with a collagen scaffold seeded with autologous cells. Indeed, the concept demonstrated new frontiers for patients and promised implementation of this solution in clinics. The bladder dome-shaped collagen scaffold presented here, pre-seeded with autologous urothelial and smooth muscle cells for augmentation, seemed to be a viable solution. Following 46 months of follow-up, including a series of urodynamic tests, the bladder function was good, with reliable results. Thanks to this seemingly successful study, the attention of urologists and investors was turned toward the tissue engineering industry. Unfortunately, the study was not continued, and the urological community was left with unanswered questions, mainly regarding the durability of this management and long-term complications. The study by Atala et al. was a spark to start Tengion, a company that aimed to fabricate commercially available electrospun biomaterial scaffolds for universal urinary tract wall replacements. Neo-Urinary Conduit and Neo-Bladder patches were evaluated during phase I and phase II clinical trials, respectively (ClinicalTrials, 2004) (Joseph et al., 2014). Unfortunately, none of these products met expectations and successfully completed the trials, and the reasons for failure were sparingly discussed to define objectives for therapy improvement. Disclosure of Tengion graft behavior and remodeling *in vivo* after implantation to patients would be particularly valuable to set future research directions. Similarly, the results of an artificial urinary conduit study were not made publicly available. Tengion biomaterial most likely failed to prevent fibrotic reaction and scarring, resulting in gradual loss of initial elasticity and compliance necessary for integration with the urinary tract. Insufficient angiogenesis within the graft likely led to hypoxia-related fibrosis. Since 2014, no registered study has planned to evaluate new biomaterials or cell-seeded grafts for tissue-engineered urinary diversion.

#### 3.1.2 Urethra

The field of urethral reconstruction, contrary to the suspicious nature of this paragraph, is so far from being the most solid argument for tissue engineering supporters. There are eight reports available, involving 180 patients who underwent urethra reconstruction procedures using tissue-engineered grafts (Barbagli et al., 2018) (Romagnoli et al., 1990) (Romagnoli et al., 1993) (Bhargava et al., 2008) (Raya-Rivera et al., 2011) (Lazzeri et al., 2014) (Ram-Liebig et al., 2017) (Osman et al., 2014). The histological structure of the urethra is less complicated than the bladder, and there is not a complex functional background. Nevertheless, in terms of urethral reconstruction, the major challenge concerns fibrosis within the graft’s lumen, averaging 8 mm–9 mm or less, which is responsible for stricture recurrence (Mangera et al., 2010).

In recent years, MukoCell^®^, a personalized tissue-engineered autologous graft, has gained much attention from the reconstructive urology community due to several accomplished clinical studies. MukoCell^®^ is a laboratory-grown graft from cells of the oral mucosa used in the treatment of urethral stenosis. (Barbagli et al., 2018) (Lazzeri et al., 2014) (Ram-Liebig et al., 2017). Reported results underlined that the application of MukoCell^®^ for urethroplasty guaranteed similar success rates to the native buccal mucosa. It must be admitted that the harvesting of a patient’s buccal mucosa epithelial cells during an off-patient clinic procedure to create a transplantable graft is the quintessence of tissue engineering management. Nevertheless, the enthusiasm should be tempered to allow a reliable assessment of this technology. The major question mark arises due to the homogeneity of the available reports, which are continuously derived from the same centers involved simultaneously in MukoCell^®^ commercialization. This product was not evaluated in large-scale trials by independent research teams. Moreover, there is limited available data transparently demonstrating the MukoCell^®^ preparation method and graft safety (one poster) (Lazzeri et al., 2014). A relatively short 12-month follow-up used in all MukoCell^®^ trials raises doubts for reconstructive surgeons in terms of therapy efficiency and superiority over standard management. In regards to the pathophysiology of wound healing after biomaterial implantation, this period is not enough to document graft resistance to inflammatory or fibrotic narrowing (Anderson et al., 2008). Delayed response to implanted biomaterials lasts up to 24 months after the initial procedure, and during all this time, slowly progressive scarring occurs (Morris et al., 2017).

Current urethroplasty techniques based on buccal mucosa are effective treatment modalities, so why does current urology need alternative materials for reconstruction? One of the reasons is the fast depletion of treatment methods for challenging cases, recurrent stenosis, and pediatric hypospadias. These patients are in real need of important therapeutic advances in segmental urethral replacement. El Kassaby et al. (2008) demonstrated in a randomized trial that the use of tissue-engineered human bladder acellular matrix (BAM) was a viable option for complex anterior urethral repair (El Kassaby et al., 2008). The leading concept of this study was to create an off-the-shelf biomaterial, an acellular product intended to become an alternative for buccal mucosa. Thirty patients underwent urethroplasty with BAM due to stricture lengths ranging from 2 cm to 18 cm and were followed for 36 months. The regeneration of the urethral wall was believed to start from native mucosa, whereas BAM was planned to generate an excellent environment for neo-tissue formation. The authors did admit, however, the lower success rate in the BAM group, especially in patients who had previously undergone interventions. This was an important observation that documented the inferiority of the acellular strategy and mostly depended on the urethral mucosa epithelial cells’ ability to populate the scaffold and reconstitute the regrown consistent layer. Following that, the same center published an observational study with five boys evaluating grafts made of tabularized poly (glycolic acid) (PGA) seeded with autologous urothelial and smooth muscle cells (Raya-Rivera et al., 2011). Tissue-engineered urethras, as described by the authors, were used for segmental urethroplasty, which is the most demanding technique due to the high failure rate in urethral surgery. In this setting, the uroflow analysis showed unobstructed urine flow up to 72 months after surgery. The costs of the treatment were not revealed. Despite the great success, the clinical value of research based on a few cases is rather low due to the inability to prove the superiority of the novel, more expensive method over standard management. Moreover, a description of the applied methodology would be difficult to comply with in other centers willing to test this option. The unspoken issue was the potential impact of a patient’s young age on therapeutic outcome. Human tissue’s ability to regenerate declines with age due to the loss of stem/progenitor cell function (Sousounis et al., 2014). Therefore, the outcomes of the methods involving individual regeneration potential might be anticipated to be worse in adults.

#### 3.1.3 Urinary incontinence

Tissue engineering efforts have concentrated on therapy for stress urinary incontinence (SUI) in women for many years. The experimental restoration of a damaged urethral sphincter has been carried out by cell transplantation of autologous myocytes, muscle-derived stem cells, and adipose tissue-derived stem cells (Pokrywczynska et al., 2016). Although similar cell populations are applied, methodologies and data acquisition are heterogeneous, making a comparison of results and choosing an adequate technique difficult. Despite these inconsistencies, available clinical trials showed that cell-based therapy had a high success rate in SUI treatment (Cornu et al., 2014) (Kuismanen et al., 2014). Most of the available trials presented short-term benefits regardless of the material used, including placebo saline injections. A thorough analysis of the data indicated that cell-based therapy did not turn out to be effective in clinical practice and should not be recommended to patients. Most of the available trials reported a short-term benefit corresponding to the effectiveness of placebo saline injections. Endpoints were based on subjective parameters, usually non-validated life-quality questioners. Interestingly, none of the studies documented using the cough test as one of the tools for evaluating SUI after therapy. As is commonly demonstrated, an increased urethral pressure profile must be interpreted with caution due to the uncertain diagnostic resolution of this method. The issue hardly ever deliberated in the studies’ “discussion” paragraph is the demarcation between improvement mediated by the bulking effect and recovery of sphincter function due to alleged induced regeneration (Vinarov et al., 2018).

#### 3.1.4 Ureter

To our knowledge, there is not any study evaluating the tissue engineering approach for ureter replacement/reconstruction in a clinical trial.

### 3.2 Costs of tissue engineering research

The global tissue engineering market is categorized into therapeutic products, tools, banks, and services. The manufacturer price of tissue‐engineered products ranges between US$ $18,950 and US$ 93,432 on average (Schneider et al., 2010). The major segments of tissue engineering products include cell therapy, gene therapy, and tissue replacement. It is estimated that the global tissue engineering market will exceed US$ 94.7 billion in the near future, with a CAGR of 23% (MarketWatch, 1997). Recognizing this potential, the National Institutes of Health (NIH) of the United States invested an estimated $940 million in regenerative medicine research in 2018 alone (National Institutes of Health (NIH), 2016). Nevertheless, the optimistic data does not reflect the significance of tissue engineering therapies in clinical practice. At this point, it must also be underlined that MukoCell^®^ is the only tissue-engineered product for the urologic patient. One of the important factors hampering the translation of experimental tissue engineering therapies is the high cost of treatment in comparison to the potential results. All studies evaluating tissue engineering therapies for urethra or urinary incontinence had a power of less than 0.8 (Vickers and Sjoberg, 2015). As a consequence, underpowered studies cannot convince medical care authorities to fund and widely implement this approach in clinics. Nowadays, considering the lack of research evidence, the question arises whether tissue engineering in urology is economically viable. In our opinion, this technology should be at the initial stage of development, and the focus should be on the confirmation of its effectiveness rather than delivering case reports. Another priority is to reduce efforts and concentrate on the pharmacoeconomic aspects of tissue-engineered procedures. Investigators should critically evaluate what effects their research will have on their future market and whether an established company would welcome a new product. Startup companies have options as well. During the initial investigation, small markets may not seem appealing to entrepreneurs (Bayon et al., 2015). However, treatment with unorthodox, technologically advanced therapies makes it more feasible to enter the smaller markets first. The best example is the success of MukoCell^®^ in reconstructive surgery of the urethra, rather than being a niche field of tissue engineering application. Apart from its effectiveness, it is used in clinical practice daily, and supporters of this method are experts in the field.

The cost of funding and discovery of tissue engineering products is primarily supported by small-to medium-sized companies in collaboration with university units receiving government or private research grants. Big pharma companies are gradually increasing their investments in tissue engineering, including urology, but it is still in its early stages, and the amount of funding available has thus far been crowded out by their near-term revenue priorities (Statnews, 2019). If tissue engineering is to revolutionize medicine, particularly urology, the disproportionately distributed funding sources must be rearranged. Careful consideration should be given to new funding models and tax incentives that will attract new sources of capital for interdisciplinary research groups combining biotechnologists, doctors, and biomaterial experts (Adamowicz et al., 2017a).

### 3.3 Law procedures

**FIGURE 3:**
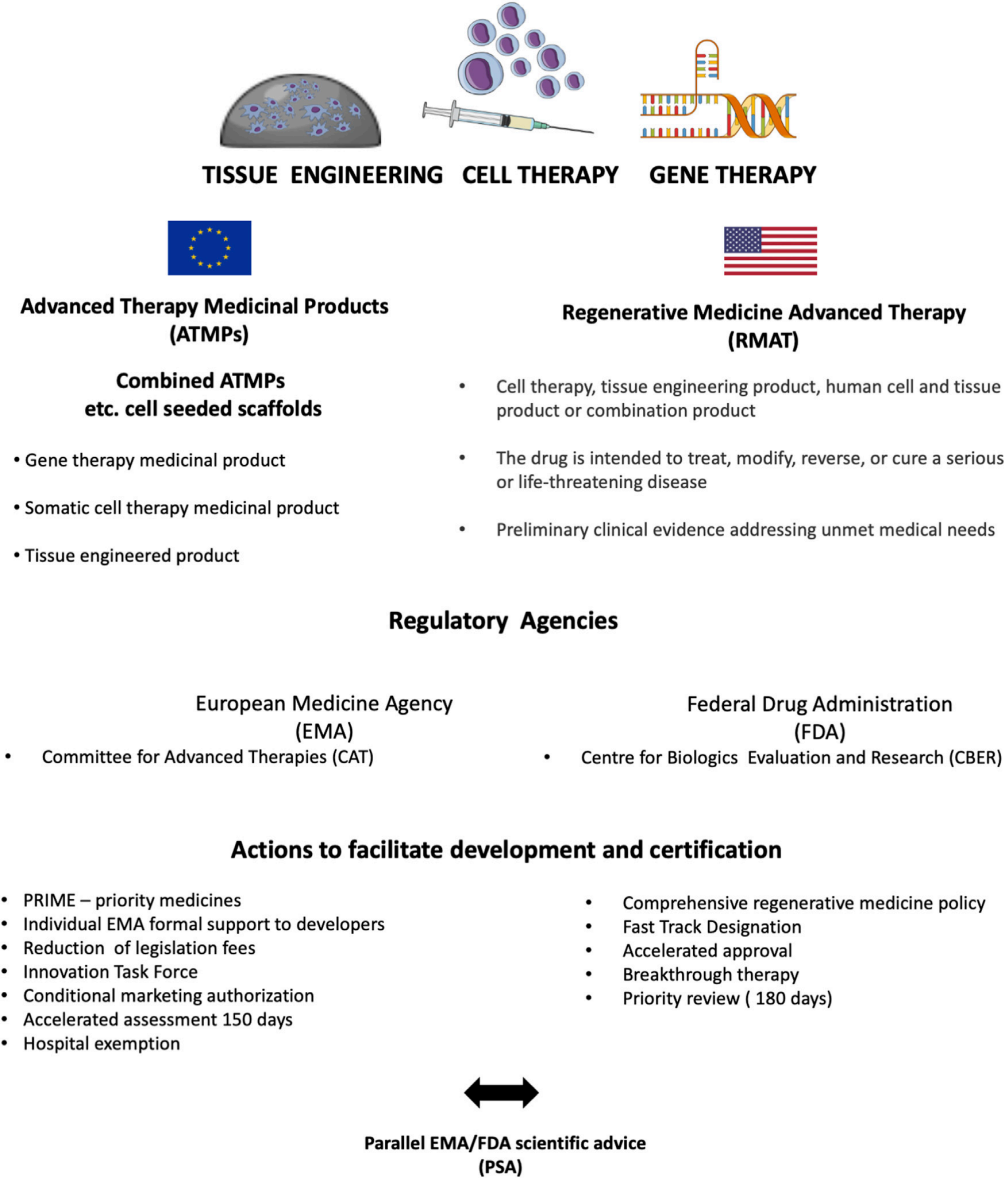
Comprehensive overview of tissue-engineered product certification in the EU and the United States.

Cutting-edge tissue engineering therapies that combine living cell transplantation with biomaterials are among the most complicated in terms of clinical trials, regulations, and the field of medicine. Tissue Engineered Medical Products (TEMPs) intended for tissue repair, and replacement are qualified as Cell-based Medicinal Products (CBMPs) or Advanced Therapy Products (ATPs) (EMEA/CHMP, 2006) (Johnson et al., 2011). In the USA and Europe, the approval criteria for TEPs are regulated by the FDA and the European Medicines Agency (EMA), respectively (Figure 3) (U.S. Food and Drug Administration, 2019) (European Medicines Agency, 1995). In contrast to the national regulatory framework, a different policy is applied to the regulation of cell therapy products to be marketed in the countries of the EU. Accordingly, there is one centralized procedure across the countries of the EU. Analogously, in the United States, the Center for Biologics Evaluation and Research (CBER) regulates cellular and biomaterial-based therapies as a part of the FDA (Center for Biologics Evaluation and Research (CBER), 2022). Good Manufacturing Practice (GMP) guidelines establish quality control standards for the manufacturing of ATMPs and are international, comprehensive, and mandatory to follow (Greenberg-Worisek et al., 2018). To date, only a few TEMPs, mostly from the field of oncologic hematology have been approved by the FDA and EMA (O’Donnell et al., 2019). It also reflects the unbalanced readiness of different branches of medicine to apply these therapies in clinical practice. In contrast to hematological departments, urological departments lack interdisciplinary equipment for *in vitro* cellular manipulation. The only solution is to outsource the manufacturing process of TEMPs and deliver them “ready to use”. MukoCell ^®^ successfully implemented this strategy. A significant disadvantage of the outsourcing strategy, if widely used, is the possibility of a loss of integrative supervision by clinicians throughout therapy and the risk of quality issues. Another option is to establish universal biotechnological units within organizational structures of leading healthcare providers to administer, manage, and serve as a local advisory board for TEMPs. Moreover, the complexity and fragility of TEMPs with regard to their vitality necessitates the education of clinical personnel to obtain a background in stem cell biology and biomaterial science.

The development of TEMPs demands the integration of stem cells, biomaterials, and chemicals, making it exceptionally challenging for market authorization and commercialization. Researchers often adopt the wrong strategy when they plan to spin out a product from their laboratory. Specifically, they start to approach regulatory issues after the novel product is at the end of its development phase. While they should plan a regulatory path at the beginning of their project, this model of research management is already encouraged by authorizing agencies. The EMA provides an expert panel to categorize and select the best regulatory pathway for novel TEMPs (TEMPs, 1995). (TEMPs, 1995) (TEMPs, 1995) (TEMPs, 1995)For instance, tissue-engineered Xenograft tissues are qualified as device biologics by the EMA instead of TEMPs. As a result, they go through a simplified certification process (European Medicines Agency, 2011). Choosing components according to regulatory guidelines at the inception of research is an underestimated factor that has a crucial impact on authorization procedures during preclinical and clinical trials.

Potentially, a wide range of TEMPs dedicated to reconstructive urology will emerge as science evolves. Realistically, clinicians, including urologists, are not trained to overcome the complex aspects of clinical effectiveness and safety, along with the regulatory and ethical issues of TEMPs. This situation may raise uncertainty and cause the reluctant introduction of innovative therapies into clinical practice. To increase the likelihood of clinical success, it is high time to gradually familiarize clinicians with the regulatory bases of therapies that are based on genes, tissues, or cells. The education of clinicians will positively influence their involvement in preclinical trials. By implementing developments in TEMP made recently by medical authorities, investigators can reduce the gap between the bench and the patient.

## 4 Achievements–Critical evaluation

### 4.1 Urothelial layer regeneration

Regeneration of the urothelial layer of the reconstructed urethra, urinary bladder, or ureter determines the reconstitution of the barrier against urine. Table 1 shows the achievements in urothelial layer regeneration. Against the dogma of reconstructive urology that urine acts as a source of nutrients for regenerating the urothelial layer, it has a devastating effect on exposed cells without the protection of the umbrella cell layer (Adamowicz et al., 2012). Difficulties in eliminating urine contact with the regenerating area are one of the reasons for the enhanced fibrotic response. Urothelium, like all epithelia, has an intrinsic tendency to spontaneously regrow from the surrounding tissue and to reform a stratified layer (Kloskowski et al., 2017). Future tissue engineering therapies will require technology to obtain a sufficient number of urothelial cells (UCs) *in vitro,* ready for transplantation or graft creation. UCs can be isolated from the native urothelium, requiring open or endoscopic surgery for tissue sampling (Kloskowski et al., 2014). There is no clinically validated protocol determining how much urothelial tissue is needed or from which urinary tract region the tissue should be harvested. In the case of invasive procedures, such protocols must be formulated to be obligatorily followed. In October 2018, the clinical trial “Urothelium Tissue Engineering Using Biopsies from Transurethral Resection of Prostate” was launched, and its results might serve as a base to define guidelines optimizing the creation of autologous tissue-engineered urothelium (J.-L. L. Nicolas Berte, 2018).

**TABLE 1:** Achievements in urothelial layer regeneration.

Study	*In vitro*/*In vivo* study	Used cell type	Achievement
Nagele et al., (2008)	*in vitro*	Human UCs	Human UCs isolated from bladder washings were used to make urothelial sheets
Lu et al., (2019)	*in vitro*	Pluripotent stem cells identified in urine	Identifying pluripotent stem cells in urine that might also belong to mesenchymal stromal cells - like progenitor cells that reside within the bladder wall
Wan et al., (2018)	*in vitro*	Urine-derived stem cells (USC)	Obtaining a differentiated epithelium of the urinary tract from USC.
Tian et al., (2010)	*in vitro*	Bone marrow mesenchymal stem cells (BMSCs)	Urothelium-like cells derived from human BMSCs can be used as an alternative source of cells for urinary bladder reconstruction
Inoue et al., (2019)	*in vitro in vivo*	UCs	First successive transplant of urothelial cells into the bladder lumen
Suzuki et al., (2019)	*in vitro*	Pluripotent stem cells (hiPSCs)	Established a protocol for the directed differentiation of hiPSCs into stratified bladder urothelium

An alternative solution was proposed earlier by Nagele et al. (2008) who showed that human UC cultures could be efficiently established from bladder washings. Stratified cultures and detached sheets stained 100% positive for pan-cytokeratin and CK20, indicating differentiation into superficial cells. Notwithstanding this fact, Uroplakin III expression, which is a specific marker for umbrella cells, was not observed. Cell sheet viability was confirmed by rapid cell outgrowth in explant cultures. An important advantage of this study was the exclusion of contentious p63-positive mesenchymal cells. Therefore, UCs obtained *in vitro* in this setting originated from precursor cells or activated mature UCs. This study raised many questions regarding the efficiency of the presented method and the quality of urothelial cells used to establish primary cultures.

In most of the diseases requiring urinary tract reconstruction, autologous urothelial cells are not suitable for application (Drewa et al., 2012). The cardinal contradiction is urothelial cancer and the related risk of panurothelial disease. Presently, there is no available method to identify and separate cancerous cells. Additionally, even in the case of benign conditions needing reconstruction, autologous cells might be compromised. For instance, the urothelium derived from obstructed urinary tracts or after chronic infections showed a lower survival rate and potential to replicate (Lee et al., 2016). When healthy urothelium is not available, stem cells or even dedifferentiated mature cells may be used as an alternative cell source.

USCs are currently being explored as a natural, accessible source for UC generation to be used in tissue engineering. USCs are a heterogenous cell population without a clear isolation methodology, and further segregation is required to distinguish the most useful subpopulations. There are also concerns regarding available isolation protocols, i.e., repeatability and efficiency (Kloskowski et al., 2015b). In general, USCs are described predominantly as pluripotent stem cells supposedly derived from renal epithelium due to high gene expression for kidney cortex markers (Pavathuparambil Abdul Manaph et al., 2018). According to a recent study by Lu et al. (2019) pluripotent stem cells identified in urine might also belong to a newly described mesenchymal stromal cell-like progenitor cell that resides within the bladder wall. Wan et al. (2018) reported that urothelial-conditioned medium combined with dynamic flow culture-induced human USC differentiation into UC with a developed uroplakin protective barrier. Differentiated USCs expressed urothelial-specific transcripts and proteins (Uroplakin III and Ia), epithelial cell markers (CK20 and AE1/AE3), and tight junction markers (ZO-1, ZO-2, E-cadherin, and Cingulin). Also, obtained UC grown on collagen matrix spontaneously formed a multilayer structure corresponding to a healthy urothelial layer.

Various types of mesenchymal stem cells (MSC), including bone marrow-derived or adipose tissue MSC, showed a capacity to induce urothelial cell differentiation (Xiong et al., 2015). Tian et al. (2010) postulated that epidermal growth factor, platelet-derived growth factor-BB, transforming growth factor-beta1, and vascular endothelial growth factor were crucial to initiate and maintain the urothelial differentiation pathway. Biotechnology is nowadays taking the first steps to develop scientific know-how in terms of controlled *in vitro* differentiation of adult cells into different cell lineages, including the urothelium. Inoue et al. recently demonstrated a method to generate human urothelial cells from dermal fibroblasts by transducing genes for four transcription factors, FOXA1, TP63, MYCL, and KLF4 (FTLK) (Inoue et al., 2019). Innovative gene engineering resulted in the creation of a cell population expressing urothelium-specific markers and capable of forming an impermeable barrier. These cells were also evaluated *in vivo* after being transplanted into the bladder of mice with Interstitial Cystitis (IC), and histological analysis revealed a significant improvement in the quality of the urothelial layer.

The discovery of iPSCs opens new perspectives for personalized regenerative medicine, as these cells may act as an unlimited source of autologous engineered tissue. iPSCs are a type of pluripotent stem cell that can be generated directly from a somatic cell. iPSCs have generated a lot of interest in tissue engineering because they can multiply indefinitely and also transform into all types of cells in the body. Suzuki et al. developed a protocol for the directed differentiation of hiPSCs into stratified bladder urothelium through the definitive posterior endoderm and caudal hindgut by recapitulating embryogenesis by using high inhibitory doses for the enzyme GSK-3 (Suzuki et al., 2019). This method was the first to obtain terminally differentiated UCs expressing uroplakins from iPSCs.

### 4.2 Regeneration of the smooth muscle layer

The development of technology for constructing tissue-engineered muscle layers is required for the proper function of the reconstructed urethra, urinary bladder, and ureter. These hollow organs have active and passive diameter tension that is controlled by a smooth muscle layer tone (Ajalloueian et al., 2018). Smooth muscle from the bladder and ureter displays patterns of spontaneous contractile activity determining the storage and voiding phases of the micturition cycle (Brading, 2006). Interestingly, even the urethral muscle layer is involved in humans in the generation of voiding pressure. Detrusor smooth muscle cells experience a sevenfold length change and continually preserve the ability to contract and generate urine outflow over this broad length range (Tuna et al., 2012). The anisotropy and heterogeneity of the mechanical characterization of the human urinary bladder are related to its complicated geometry, structure, and functions. Unfortunately, tissue-engineered smooth muscle layers with a similar function have yet to be replicated. Table 2 shows the achievements in regeneration of the smooth muscle layer.

**TABLE 2:** Achievements in the regeneration of the smooth muscle layer.

Study	*In vitro*/*In vivo* study	Used cell type	Achievement
Opitz et al., (2007)	*in vitro*	Vascular SMCs	Determination of phenotypic plasticity of vascular-derived SMC which allows oscillation between proliferative and differentiated phenotype depending on pressure stress conditions
Pokrywczynska et al., (2019)	*in vivo*	Adipose tissue-derived mesenchymal stromal cells (ASCs)	ASCs create an environment rich in morphogenetic signals corresponding to an early organogenesis environment when the mesenchymal forms an early smooth muscle layer
Liu et al., (2017)	*in vivo*	USCs	The use of a porous SIS scaffold seeded with USCs allows for the regeneration of the urethra
Mirzaei et al., (2019)	*in vitro*	Human iPSCs	Human iPSCs seeded on poly (lactic-co-glycolic acid) (PLGA) resulted in acquiring a smooth muscle
Hoogenkamp et al., (2016)	*in vitro*	UCs, SMCs	Development of a method of obtaining collagen scaffold of the size of the entire urinary bladder
Ardeshirylajimi et al., (2018)	*in vitro*	MSCs	The scaffold releasing TGF-β and seeded with MSCs allows for bladder regeneration

Opitz et al., (2007) demonstrated the phenotypic plasticity of vascular-derived SMC, and their ability to oscillate between a proliferative and differentiated phenotype in response to pressure stress conditions. This phenomenon might be an essential clue in efforts to regenerate human detrusor muscles, as it shows the necessity of stimulating regenerating tissue through physiological pressure stress. This relationship must be kept in mind because, in urology, there is a tendency to preserve long-lasting decompression of healing regions by catheterization.

The restoration of the smooth muscle layer for tissue engineering purposes might be accomplished by induced regeneration combined with the adaptive cytoskeletal plasticity of spontaneously regenerating cells. This mechanism is dominant in cases where part of the native urinary tract wall acts as a regeneration primer and is a source of muscle precursor cells (Liu et al., 2019). Nevertheless, the self-regenerating capability of the smooth muscle layer is minimal, therefore boosting endogenous regenerative potency is necessary (Drewa et al., 2008).

MSC transplantations were documented to stimulate the regeneration of tissue-engineered urinary bladders by activating the hedgehog signaling pathway. Pokrywczynska et al. (2019) showed, using a rat model, that MSC created an environment rich in morphogenetic signals corresponding to an early organogenesis environment when the mesenchyme forms an early smooth muscle layer. In this scenario, MSC shaped the paracrine framework to support the regrowth of the muscle layer, guided by activated muscle cells sprouting from the entire region. Adult smooth muscle cells retain the ability to form a subpopulation of highly proliferative precursor cells. Yang et al*.* formulated a similar conclusion using mesenchymal USCs in a rabbit urethroplasty model to trigger regeneration within the SIS graft. Part of the PKH67 labeled USCs were capable of differentiation into cell lineages expressing urothelial, smooth muscle, endothelial, and interstitial cell markers, proving the multipotency of this cell population. Application of USC led to the development of a low-grade inflammatory response with the extensive rebuilding of the muscle layer expressing myosin and actin (Liu et al., 2017).

The majority of indications for urinary tract reconstruction include cancer, which eliminates the possibility of applying autologous cells harvested from urinary tracts due to safety concerns. For this purpose, Mirzaei et al. (2019) investigated human iPSCs as a stem cell source for the generation of bladder smooth muscle cells. They reported that *in vitro* cultivation of iPSCs seeded on PLGA resulted in acquiring a smooth muscle phenotype confirmed by the expression of alpha-smooth muscle actin (ASMA), smooth muscle 22 alpha (SM- 22a), calponin-1, caldesmin1, and myosin heavy chain (MHC). It was discussed that the nanofibrous scaffold resembled native bladder ECM and provided adhesive signaling that enhanced differentiation. The characteristics of the attached phenotype might indicate immature smooth muscle cells or myofibroblasts. These cells might not accomplish the development of an adult SMC phenotype that determines complex function and hierarchal organization. The phenotypic changes during the smooth muscle differentiation pathway have actin and myosin appear early in development, whereas caldesmon and calponin serve as markers for the final smooth muscle differentiation stages (Huber and Badylak, 2012). Yipeng et al. (2017) underlined that the acquisition of a smooth muscle phenotype depended on a 3D scaffold and could not be replicated in a 2D culture environment. 3D-structure and the geometry of the extracellular matrix modulated SMC behavior and the development of the contractile apparatus. These interactions, which are currently being gradually identified and described, are particularly crucial for the restoration of the linear arrangement of regenerated smooth muscle layers within urinary tracts.

Priority is given to maintaining control over the arrangement of SMCs cultivated on scaffolds for graft preparation by guiding them using a configured biomaterial ultrastructure. The rationale behind it is that an uncontrolled increase in ECM anisotropy might initiate cancerogenic transformation (Walker et al., 2018). Hoogenkamp et al. (2016) presented a method to obtain a collagen scaffold in the shape of the whole bladder with integrated anastomotic sites for the ureters and urethra. An integrative approach for the restoration of ECM continuity from the ureters to the urethra is an idea worth further development as it should improve function recovery by reconstituting the consolidated middle layer of urinary tracts. The fabricated scaffolds’ ultrastructure had a uniform wall thickness and a unidirectional pore structure to facilitate the cell’s migration and attachment. The polarized cross-section of the scaffold wall with uniform wall thickness and a unidirectional pore structure guided proliferating SMCs and allowed for the restoration of layered architecture.

The smooth muscle layer regeneration *in vivo* is a time-consuming process, while most of the regulating growth factors and cytokines are rapidly localized and systemically eliminated (Khosravi et al., 2007) (Lee et al., 2011). This is one of the reasons why the initial satisfactory regenerating results diminish over time after the initial procedure. Ardeshirylajimi et al. recently demonstrated the scaffold’s ability to release TGF-β in a controlled manner to direct MSC differentiation into smooth muscle (Ardeshirylajimi et al., 2018). Additionally, the 3D network of electrospun nanofibers enabled MSC parallel alignment, which successfully enhanced the formation of muscle bundles. A novel biomimetic scaffold provided multidirectional stimulation of the cellular component to induce the desired differentiation pathway. The sparingly discussed issue is the influence of the short shelf-life of most of the growth factors and cytokines used for the induced regeneration of SMCs on the eventual market optimization of bioactive biomaterial scaffolds (Suarato et al., 2018). Currently, fabrication of a ready-to-use scaffold, e.g., a bladder patch enriched with growth factors, would be challenging due to difficulties in the development of a reliable method for bioactive component preservation.

### 4.3 Regeneration of bladder innervation

It has been proven that tissue engineering methods enable the regeneration of the urothelial epithelium, smooth muscle, and blood vessels in the reconstructed bladder. The problem remains to recreate the neural network as shown in Table 3. The proper functioning of the bladder is based on the cooperation of all layers of the bladder wall. This allows the holding and passing of urine to be regulated.

**TABLE 3:** Achievements in the regeneration of bladder innervation.

Study	*In vitro*/*In vivo* study	Used cell type	Achievement
Madduri et al., (2009)	*in vitro*	Dorsal root ganglion (DRG)	Glial cell line-derived neurotrophic factor (GDNF) significantly influences axonal elongation, and nerve growth factor (NGF) induces extensive axonal branching
Kikuno et al., (2009)	*in vivo*	**-**	NGF may support the regeneration of a functional bladder formed from BAM
Nitta et al., (2010)	*in vivo*	Skeletal muscle-derived multipotent Sk-34 and Sk-DN stem cells	The transplant allowed for significant functional recovery (80%) thanks to the incorporation of the transplanted cells into the damaged peripheral nerves and blood vessels
Adamowicz et al., (2011)	*in vitro*	Schwann cells	Protocol for the effective isolation of Schwann cells from a pre-degenerating peripheral nerve

To achieve this, it is important to innervate the tissue-engineered bladder. The process consists of the following steps: axonal outgrowth, neural survival, branching, and target nerve reconnection (Sharma and Bikramjit, 2022). Bladder reinnervation is a complex process, therefore many studies are in the initial stages of research.

Madduri et al. (2009) showed that the combination of GDNF and NGF has a positive effect on the recovery of injured peripheral nerves. GDNF significantly influences axonal elongation, and NGF induces extensive axonal branching. Kikuno et al. (2009) however, showed that VEGF combined with NGF allows for the formation of aggregated bundles of smooth muscles and the regeneration of nerves and fibers.

An alternative solution is to use cell-based therapies. Nitta et al. used skeletal muscle-derived multipotent stem cells, which after transplantation differentiated into Schwann cells, perineurial cells, vascular smooth muscle cells, pericytes, and fibroblasts around the bladder. The applied method allowed for the recovery of 80% of the bladder’s functionality (Nitta et al., 2010). Another approach is to use isolated Schwann cells to innervate the tissue-engineered bladder. Adamowicz et al. (2011) developed a protocol for the effective isolation of Schwann cells from pre-degenerating peripheral nerves, which can deliver the required amount of cells for transplantation into a urinary bladder graft.

### 4.4 General concerns related to cell-based tissue engineering

The clinical application of cell-based therapies in tissue engineering is indispensable, but there is also a significant question that is hardly ever addressed. First of all, oncological safety of potential therapy utilizing differentiated *in vitro* cells. Every cell division has a small chance of introducing deleterious mutations, and systemic mechanisms such as immune recognition aimed to correct these alternations do not function in *in vitro* culture (Närvä et al., 2010). Some reports indicated that the tumorigenicity of stem cells had been predicted to increase proportionally with the length of *in vitro* culturing (Knoepfler, 2009).

In most cases, the final recommendation of the experimental method to be applicable for differentiation into SMCs or UCs was based on the successful identification of several essential markers. It must be admitted that knowing the complexity of urothelium or detrusor muscle function and cytoarchitecture, this reasoning is an oversimplification. In fact, these cells might be considered a rather immature cell population with an unstable phenotype. Detected markers might only be a manifestation of uncontrolled or partial activation of the differentiation pathway, and their presence does not determine function like healthy bladder SMCs or UCs.

Moreover, the incomplete differentiation process supported by indiscriminately activated pathways might become a platform for malignant transformation. Unfortunately, we are also doing little to advance our understanding of cell differentiation. Current research accentuates that the differentiation fate of UCs or SMC precursors is dependent on paracrine stimulation and biomechanical signaling derived from the scaffold structure. The next step is going to be genetic engineering aimed at triggering the desired phenotype. Zhao et al. obtained SMC expressing a mature contractile phenotype from USCs using both miR-199a-5p and TGF-β1 (Zhao et al., 2019). The results of the study showed that SMCs converted by miR-199a-5p and TGF-β1 had significantly better contractile activity than TGF-β1 alone.

Another problem is the survivability of transplanted cells within the regenerating environment. There is a rising number of reports indicating that transplanted cells, either alone or as part of tissue-engineered grafts combining biomaterials, are eliminated mainly by macrophages within several weeks (Arutyunyan et al., 2015) (Yang et al., 2008). In this situation, implanted cells, i.e., MSC, act as a temporary booster, modifying the regenerative environment but without long-lasting effects (Pokrywczynska et al., 2018). Considering this, the ability to rebuild neo-tissue that is integrated with host urinary tracts by implanting mature cells is to be questioned. Alternatively, according to some authors, the putative regenerating effect of transplanted stem cells depends on infiltrating immune cells (Abnave and Ghigo, 2019).

## 5 Biomaterials

The extracellular matrix of the urinary tract wall is a three-dimensional network composed of multi-domain macromolecules such as collagen, elastin, glycosaminoglycans (GAGs), and cell-binding glycoprotein (Binette and Binette, 1991). Successful tissue-engineered-based replacement of the urethra, urinary bladder, or ureter requires the development and fabrication of a biomaterial scaffold acting as a supporting frame for growing tissue.

### 5.1 Urethra

Acellular biomaterial scaffolds are desired for implantation as they do not require the expensive and time-consuming process of cell seeding. From the perspective of commercialization of tissue engineering therapies, the acellular approach is more accessible due to the convenience of use in urology departments that are not equipped with the infrastructure necessary for cell culturing. It is generally believed that cell-free biomaterials are suitable for small defects only, but to our knowledge, there is no study comparing cell-seeded and acellular scaffolds for urethroplasty outside of a control group (Versteegden et al., 2017).

In the pioneering study of Dorin et al. (2008) the authors used acellular bladder submucosa for tabularized urethroplasty at varying lengths in a rabbit model. They reported breakthrough observations for further research focusing on urinary tract wall replacement, including the urethra. Bridging grafts showed ingrowth and healthy regeneration of the urethral wall only at the anastomotic edges. At the same time, increased collagen deposition and fibrosis toward the center occurred. Orabi et al. (2013) demonstrated in a preclinical study evaluating tabularized collagen scaffolds for extended urethral defects using a canine model, that cell seeding is necessary to counteract the fibrotic reaction and stricture formation.

These observations led to the evolution of regenerating biomaterials into the design of bioactive matrices. Accordingly, Jia et al. (2015) applied collagen membranes linked with VEGF for the repair of 5 cm-long anterior urethra defects using a canine model. The study’s concept concentrated on the potential ability of VEGF to improve neo-angiogenesis and related blood supply within the implanted biomaterial. The authors concluded that collagen scaffolds enriched with VEGF promoted urethral tissue regeneration and improved the function of the neo-urethra. Non-etheless, the attached histological data documenting the regrowth of the urethral wall demonstrated a highly disordered network of neo-vessels and smooth muscle cell bundles that deviated significantly from normal muscle architecture. It is very likely that after a follow-up longer than six months, the stricture would be rebuilt due to the gradual increase of local ischemia and a lack of spatial resistance usually mediated by the resting tone of muscle layers.

Pinnagoda et al. (2016) delivered data from the longest reported follow-up after partial completion of urethral replacement with a tissue-engineered acellular graft. In this study, the rabbit urethra was reconstructed with a double-layer collagen scaffold expected to support regeneration and simultaneously prevent the graft from collapsing under the pressure generated by forming a scar. After nine months, the histological analysis revealed a well-regenerated urethral wall with stratified epithelium and an abundance of muscle cells. This promising result should be approached cautiously because the collagen scaffold was approximately 1.3 mm thick. In this case, the regenerating area could be passively perfused by neighboring tissue, preventing the development of ischemia. In general, 1 mm–2 mm is a limit value for efficient oxygen exchange and diffusion derived from the local blood supply (Moon and West, 2008). In terms of greater distances, the hypoxic zone promotes a fibrotic response. For a clinical application, a collagen scaffold approximately 1 mm thick does not provide efficient mechanical endurance for urethral surgery; the estimated young modulus was approximately seven kPa, which corresponds to human liver tissue. On the other hand, the mechanical properties of scaffolds created from liquid collagen are challenging to balance due to the tendency during collagen solution dehydration to form rigid structures with a high young modulus (Meyer, 2019).

Nowadays, biomaterial science still struggles to replicate the high biocompatibility of naturally derived biomaterials and implement them as a fabricated scaffold for tissue engineering. AMA is a promising biomaterial with an abundance of unique properties (low immunogenicity, promotion of epithelization, anti-inflammatory properties, angiogenic and antiangiogenic properties, antifibrotic properties, antimicrobial properties, and anticancer properties) (Adamowicz et al., 2019b). Despite excellent application potential, it remains a relatively unknown biomaterial for the urological community.

Ophthalmologists, on the other hand, routinely apply AM as a biological wound dressing for the treatment of corneal injury (Dua et al., 2004). AM grafts were utilized for urethral wall replacement in several studies using small animal models (Shakeri et al., 2009) (Güneş et al., 2017). All of them underline excellent AM properties and the ability to induce local regeneration. Nevertheless, the research data derived from these reports has low translational potential because of insufficient research group numbers, essential diagnostic tools, and the heterogenic model of urethral injury. Admittedly, two short reports show the feasibility of using AM for urethroplasty in humans (Koziak et al., 2007) (Koziak et al., 2004). Although these papers presented a novel method, the leading concept of the studies (conducted on a few patients) was instead to demonstrate extravagance surgery rather than to change the current management dependent on buccal mucosa.

An electrospinning technique provided the ability to produce biomaterials with nanoscale properties for tissue engineering. Given the importance of intercellular interactions between the biomaterial and the ingrowing tissue, electrospinning allows for the fabrication of 3D scaffolds arranged in a complex fibrous porous matrix similar to a healthy ECM (Davis et al., 2018). It is especially important for the regeneration of multilayer hierarchically organized structures, such as the urinary tract wall, as this parallel spatial architecture allows the regenerating tissue to maintain its orientation. Moreover, electrospinning allowed for the creation of small-diameter tubes with high uniaxial mechanical resistance appropriate for urethral surgery, as demonstrated by Sánchez-Pech et al. (2019) The significant advantage of their work is that it includes extensive biomechanical analysis, which is usually only provided marginally. Composite tubular scaffolds created from PCL and PLGA exhibited a high elastic modulus (19 MPa), ideal to withstand bursting pressure within the human urethra. Additionally, a low strain-to-break value should guarantee a proper surface for stable anchoring and fixing sutures, which would be necessary to convince a surgeon to use them. Notwithstanding, the optimal biomechanical features of PCL-based electrospun scaffolds have a high hydrophobicity and hence create an environment that inhibits cell attachment and growth. This is a seldom-reported problem that needs to be addressed by modification of the scaffold’s surface. Alternatively, the high adaptability of electrospinning technology makes it possible to utilize electrospun nanofibers as a skeleton for a naturally derived biomaterial with low mechanical resistance, for instance, AM (Adamowicz et al., 2016).

To overcome the mentioned problem, the application of several low-cost modes such as centrifugal jet spinning and immersive rotary jet spinning combined with hydrogels might solve the problem of an environment that impedes cell attachment and growth (Ravishankar et al., 2021), (Gonzalez et al., 2017).

Khang et al. (2017) report a procedure that allows for engineering biphasic Janus-type polymeric nanofiber networks *via* the centrifugal jet spinning technique that provides unique structural support and biological activity and has many applications in tissue engineering approaches, such as where there might be a need for different properties on either side of the scaffold, such as environment resistance on one side and biocompatibility or potential therapeutic properties on the other side.

Sharma et al. (2022) used Contact-Active Layer-by-Layer Grafted TPU/PDMS and were able to validate TPU/PDMS blends as an antiencrustation and antibacterial platform for next-generation urological biomaterials with physiological relevancy for functionality. These layer-by-layer grafted blends displayed significant grafting stability and antibacterial efficacy against common uropathogens. The application of biomaterials in tissue engineering in urethral reconstruction has been described in Table 4.

**TABLE 4:** Application of biomaterials in urethral reconstruction.

Study	Biomaterial	*In vitro*/*In vivo* study	Model	Outcome
Dorin et al., (2008)	Acellular collagen matrix	*in vivo*	rabbit	It was established that .5 cm is the maximum defect distance that can support proper tissue formation using acellular tubularized grafts
Orabi et al., (2013)	BAM	*in vivo*	canine	The use of cell-seeded tubularized urethral scaffolds allows for the repair of defects up to 6–7 cm long
Jia et al., (2015)	Collagen scaffold modified with collagen binding domain (CBD) VEGF	*in vivo*	canine	The use of CBD-VEGF allowed for a much thicker epithelial cell layer compared to the collagen group
Pinnagoda et al., (2016)	Acellular double-layered collagen scaffolds	*in vivo*	rabbit	After nine months of research, they observed significant changes in acellular double-layered collagen scaffolds, obtaining a structure similar to the normal urethral tissue
Shakeri et al., (2009)	Amniotic membrane	*in vivo*	rabbit	Urethral reconstruction was successful in all 20 operated rabbits, with no inflammation or tissue loss
Güneş et al., (2017)	Buccal mucosa and amniotic membrane	*in vivo*	rabbit	The combination of buccal mucosa and amniotic membrane for ventral onlay penile urethroplasty contributed to better tissue healing
Koziak et al., (2007)	Amniotic membrane	*in vivo*	human	Regeneration of extensive ureteral defects without serious complications

### 5.2 Urinary bladder

The urinary bladder’s proper function is dependent on its ability to continually repeat loading and unloading cycles corresponding to urine storage and voiding (Yamanishi et al., 2011). This behavior model needs biomaterial for tissue engineering purposes that can withstand dynamic pressure changes during frequent mechanical loading and unloading (Adamowicz et al., 2017b). The bulk of studies introducing new biomaterials for urinary bladder replacement was published several years ago. Nowadays, sporadically appearing in articles covering this field, the topic has a repetitive character.

In the field of urinary bladder experimental reconstruction, the polymer materials PLA, PGA, and PLGA were the most influential contributors to the creation of biodegradable cellular scaffolds (Serrano-Aroca et al., 2018). All these biomaterials have FDA approval for clinical usage, and their degradation rates can be changed based on their molecular weights and compositions (FDA’s Regulatory Science Program for Generic PLA/PLGA-Based Drug Products, 2016).

Non-etheless, the major disadvantage of biodegradable copolymers such as PLA, PLGA, and PGA is evident rigidity and non-linear elasticity in comparison to the native bladder wall (Ajalloueian et al., 2018). When using these biomaterials, micro-environmental mechanical stress may affect regenerating tissue, inducing scarring by activating stretch-induced activation of TGF-β1 (Yong et al., 2015). Baker et al. (2009) showed a correlation between biomaterials’ Young’s modulus and a scaffold’s ability to support urothelial layer formation *in vitro*. Horst et al. (2017) proposed to enrich a PLGA scaffold with polyester urethane to improve elasticity and thus adjust it to a highly compliant urinary bladder wall. Indeed, the obtained biocomposite exhibited extraordinary passive elasticity, but careful analysis of cystograms demonstrated a stepped increase in intravesical pressure. This indicated the dominance of mechanical properties related to PLGA that were particularly visible eight weeks after bladder reconstruction. It was likely a consequence of the unstable mechanical properties of electrospun polyester urethane during uncontrolled degradation in the urinary tract environment. In order to design scaffolds for urinary bladder wall replacement, the degradation period needs to be adjusted to the time-lapse of tissue regeneration to prevent structural failure of the implant and graft rupture. Research data concerning changes in mechanical parameters and structural integrity during the degradation of electrospun scaffolds for soft tissue regeneration is minimal.

Besides difficulties in the adaptation of mechanical parameters, the tissue-engineered-based reconstruction of the urinary bladder demands the fabrication of biomaterial scaffolds in sizes applicable for human use. In most of the studies, authors introduce different cell matrices utilizing small animal models using biomaterial samples without a standardized or repeatable fabrication protocol (Pokrywczynska et al., 2014). We can take a chance and say that in most cases, biomaterials tested on small animal models would be very difficult or even impossible to manufacture due to the size needed for translational animal or human trials. It is a reason why scaffolds made from decellularized extracellular matrices became popular in the field of bladder tissue engineering (Pokrywczynska et al., 2015).

BAM is derived from the bladder after a decellularization procedure conducted according to established protocols that enable the preservation of the native ECM architecture. Hence, BAM is composed of a complex collagen network enriched with fibronectin, elastin, and plenty of GAGs and growth factors (Song et al., 2013). It should be emphasized that a particular arrangement of fibril proteins presented within BAM cannot be artificially replicated by current technology. Also, BAM retains an intact basement membrane, supporting rapid re-epithelialization. The rationale for BAM matrix fabrication was the assumption that its bioactivity profile would enhance regeneration by stimulating and guiding growing cells naturally, restoring the urothelial and detrusor muscle layers. The recent study of Pokrywczynska et al. (2018) proved that BAM undoubtedly created a favorable regenerative environment, but it also exhibited the same limitations as all known biomaterials tested for bladder augmentation so far. First of all, the active regeneration evaluated as the restoration of a healthy layered bladder structure was observed only within BAM regions firmly attached to the native bladder wall. The closer to the center of the graft, the more abundant the scar tissue was and, analogously, the decrease in the density of neovessels. Similar to available reports, regeneration of the bladder wall did not occur evenly. Instead, a reconstructed part of the bladder could be divided into three regions. The part of the graft that borders native tissue with well-regrown urothelial and smooth muscles. The transient part where the regeneration quality is heterogenic, due to the gradually increasing content of fibrotic tissue disrupting the newly forming urothelial and muscle layers. Finally, the center of the graft is overgrown with thick stellar scars responsible for an average graft shrinkage of 50%. As the mechanism of scar development is strictly linked to an insufficient blood supply and related local ischemia, the counteraction might be inducing angiogenesis. For instance, Jiang et al. (2016) fabricated BAM modified with VEGF-loaded PLGA NP. The study was conducted using a rabbit model. The VEGF quantification results demonstrated that the modified BAM achieved long-term sustained VEGF release *in vivo*. The contractile rate of the acellular matrix in the experimental group was significantly lower than in the control group. The functional evaluation of isolated stripes *in vitro* revealed that bladders reconstructed with VEGF-supplemented grafts were more responsive and exhibited the ability to undergo rapid cyclic contraction and relaxation. Incorporating additional substances into biomaterials such as BAM is a common strategy used to obtain a specific effect and control the regeneration process.

On the other hand, we still do not have enough knowledge to modulate such a complicated process intentionally. Zhou et al. (2013) announced that the co-administration of PDGF and VEGF with BAM significantly improved muscle contractility and angiogenesis. There was, however, no control group with one of the growth factors to check its role autonomously. Before the introduction of regular supplementation of the regenerative environment with bioactive substances, there are several questions to answer. Firstly, at what point during the healing process does the particular growth factor act? Secondly, what concentration is required? Thirdly, what is the exposure time to obtain the appropriate effect?.

Interestingly, despite different published strategies to use growth factors to supply the regenerative environment, no research groups have discussed potential risks. For example, VEGF, which is the most often used to improve regeneration outcomes, is also involved in cancerogenesis (Takahashi and Shibuya, 2005). VEGF is one of the potent factors inducing MET in mesodermally derived neoplasms. The healing environment, especially when artificially created within biomaterials, is susceptible in the long term to cancerogenic transformation. The presence of cells at various stages of differentiation and chronic inflammation *per se* creates a favorable environment for tumor transformation. If we additionally add potent bioactive substances without a deep background understanding of their action, we may trigger cancerogenic transformation.

Biomaterials of natural origin, such as the aforementioned BAM, are often used in tissue engineering due to their very good mimicry of the natural environment for cell growth, providing adhesive substances, cell binding sites, and compatibility with the tissues surrounding the regenerated organ or tissue (Dalamagkas et al., 2016).

Aside from their many benefits, biomaterials have some drawbacks, such as the limited ability to modify them and the heterogeneity of the scaffold structure in terms of chemical purity (Schmidt and Leach, 2003). In addition, most natural polymers used in tissue engineering have limited and batch-dependent mechanical properties, which often make it impossible to unequivocally assess the effectiveness of the method used.

Synthetic biomaterials can also be distinguished, the great advantage of which is the possibility of modifying their structure during the production stage. The second most important thing is the production of identical scaffolding on a large scale, which will make it possible to reproduce the same results (Dalamagkas et al., 2016).

Sivaraman et al. (2017) highlighted the advantages and disadvantages of natural and artificial biomaterials using the example of a hydrogel, and how their combination can affect the properties of the hydrogel itself. They used composite hydrogel of Tetronic 1107-acrylate with ECM moieties like collagen and hyaluronic acid seeded with bladder smooth muscle cells, which provided a viable environment for bladder smooth muscle cells to survive and reconstruct the scaffold. In comparison with the control, acellular hydrogel, the mechanical properties, stiffness, and strength of the cellular composite hydrogels were significantly greater. The team reports that culturing the construct for longer periods after bladder smooth muscle cell seeding and adding growth factors to the composite hydrogel system would further aid in accurately mimicking the mechanical properties of the bladder wall. Furthermore, Sivaraman et al. provide evidence that Poly (Carbonate-Urethane)-Urea scaffolds possess high porosity and cytocompatibility, indicating their ability to support bladder cell growth and the formation of viable tissue. The researchers also demonstrated that the Poly (Carbonate-Urethane)-Urea scaffolds are suitable for the bladder’s primary functions of maintaining low pressure during storage and withstanding high pressure during voiding (Sharma et al., 2021).

Promising synthetic biomaterials also include graphene and graphene-based nanomaterials, which, due to their structure, have many unique properties and are especially useful in the innervation of organs reconstructed using tissue engineering techniques. The mechanical strength of most polymers is characterized by declining strength when used under physiological conditions. This unique, two-dimensional material, thanks to its organized structure, is characterized by high strength and at the same time is stretchable and elastic (Ławkowska et al., 2022).

Additionally, as reported in the literature, nanomaterials based on graphene can improve Young’s modulus and increase the compressive and tensile strength of the scaffold itself (Tiwari et al., 2020), (Cheng et al., 2018).

The most breakthrough application of graphene in reconstructive urology, however, is its use for the replacement of the neuronal network of the tissue-engineered urinary bladder.

The folded structure and adapted porosity of the biomaterial not only provide mechanical support for cell proliferation but also replace the extracellular matrix, inducing the appropriate growth and elongation of axons (Adamowicz et al., 2020) (Ławkowska et al., 2022).

A major problem with the reconstruction of the urinary bladder is the occurrence of inflammation. Here, an additional advantage of graphene is its antibacterial effect, which may additionally support the process of reconstruction of tissues and organs of the urinary system (Liu et al., 2011).

However, additional studies should be carried out to unequivocally assess the cytotoxicity of graphene-based nanomaterials and to standardize graphene production methods so that the highest quality graphene can be used in the research. Biomateirals of natural and synthethic origin in the reconstruction of the urinary bladder have been mentioned in Table 5.

**TABLE 5:** Application of biomaterials in the reconstruction of the urinary bladder.

Study	Biomaterial	Biomaterial fabrication method	*In vitro*/*In vivo* study	Model	Outcome
Horst et al., (2017)	PLGA on a BAM	A hybrid microfibrous scaffold was obtained by direct electrospinning of PLGA on a BAM	*in vivo*	rat	The seeded scaffolding ensured layered regeneration of the bladder walls *in vivo*
Pokrywczynska et al., (2018)	BAM	BAM was obtained by a multistep detergent washing procedure	*in vivo*	pig	The tissue-engineered bladder worked normally. Stem cells additionally supported the regeneration of the urinary bladder
Jiang et al., (2016)	PLGA nanoparticle-modified BAM	BAM was incorporated with VEGF and bFGF-loaded PLGA nanoparticles and mixed with a hydrophilic gel	*in vivo*	rabbit	The scaffolding developed proved to be an effective method for achieving long-term sustained release of VEGF and bFGF.
Zhou et al., (2013)	BAM	BAM was incorporated with platelet-derived growth factor-BB and VEGF.	*in vivo*	rabbit	BAM combined with PDGF-BB and VEGF significantly improves muscle contractility and angiogenesis
Adamowicz et al., (2020)	AM and graphene	Graphene layers were transferred without modifying the AM surface	*in vitro*	-	Composite made of AM, graphene, and seeded with smooth muscle cells was capable of induced contraction *in vitro*

### 5.3 Ureter

Various types of biomaterials were introduced in ureteral reconstruction as shown in Table 6. Most studies focused on acellular, tabularized SIS grafts using a porcine model (Liatsikos et al., 2001) (Smith et al., 2002). The translational potential of these reports was low due to the different methodologies used and the short follow-ups. Studies conducted using a porcine model also did not address clinical needs as model ureter injuries could be effectively treated with available surgical techniques. The primary aim for tissue engineering research should be to develop a graft suitable for ultimately bridging the long ureter gap. In addition, there is a need to reconstitute peristaltic motion, to prevent incrustation, and provide long-term patency of the reconstructed hollow region (Adamowicz et al., 2019a).

**TABLE 6:** Application of biomaterials in the reconstruction of the ureter.

Study	Biomaterial	Biomaterial fabrication method	*In vitro*/*In vivo* study	Model	Outcome
Smith et al., (2002)	Porcine SIS	A porcine SIS allograft was performed	*in vivo*	pig	The SIS transplant caused the regrowth of the ureters
Xu et al., (2012)	PLLA	The PLLA was dissolved in chloroform, cast into .8 mm-thick polymer films, and then evaporated at 25°C–28°C with a 20% relative humidity	*in vivo*	rat	The scaffold created allowed for the proper proliferation of cells and the creation of vascular networks
Shi et al., (2012)	PLA and collagen	Chloroform was used to dissolve PLA to create polymer films. The films were spiral-wrapped around a glass rod. The scaffold’s exterior surface was then covered with a layer of mesh made either entirely of PLA or PLA and collagen	*in vitro, in vivo*	mouse	Human adipose-derived stem cells (hADSCs) have been differentiated into a urothelial lineage by alternating the microenvironment with urothelial cells. The scaffold is compatible with cell survival and proliferation
Koch et al., (2015)	Extracellular matrix crosslinked with carbodiimide (CDI), genipin (GP), glutaraldehyde (GA), or glutaraldehyde (BP)	The decellularized ureters were then crosslinked with different agents, such as GP, CDI, GA, and BP.	*in vitro, in vivo*	rat	Carbodiimide crosslinked scaffolds showed multilayer formation of smooth muscle cells
Zhang et al., (2012)	Silastic tubes	Six female beagles had silicone tubes inserted into their peritoneal cavities. The tubes were removed after three weeks, and the tubular tissue that covered them was gently elongated	*in vivo*	canine	A two-month follow-up showed that the neo-ureter demonstrated normal ureteral architecture. The multilayered urothelium was surrounded by smooth muscle bundles
Liao et al., (2013)	BAM	The bladder of a rabbit was removed and decellularized	*in vivo*	rabbit	At 8 and 16 weeks after implantation, the scaffold was characterized by multilayer urothelium and organized bundles of smooth muscles
Zhao et al., (2019)	Vessel extracellular matrix (VECM)	Seeded VECM was tubularized and wrapped by two layers of a rabbit omentum for vascularization	*in vivo*	rabbit	Histological evaluation showed a layered structure of the ureter with a multilayered urothelium over the organized, smooth muscle tissue

Several studies evaluated tissue-engineered grafts made from PLLA on small rodent models. The quality of this report was low and must be critically classified due to the low number of animal groups, lack of controls, and several weeks of follow-up (Xu et al., 2012) (Shi et al., 2012).

The biocompatibility of the introduced biomaterials was not enough to prevent a fibrotic reaction and stricture formation. To overcome these problems, Koch et al. developed a methodology for manufacturing decellularized ureters obtained from pigs (Koch et al., 2015). Proper decellularization techniques enable the use of xenogeneic ECM grafts without the risk of acute rejection. The decellularized ureter was described as a thin, flexible biomaterial without resistance to collapse. This is the reason why the authors applied different crosslinking agents (carbodiimide, glutaraldehyde, and genipin) to improve its mechanical characteristics. Carbodiimide-crosslinked scaffolds increased infiltrating 3T3-cells and smooth muscle cells’ multilayer formation. Nevertheless, the prepared grafts were not evaluated *in vivo*. Evaluation of biomaterial biocompatibility by implanting biomaterials into rat subcutaneous tissue is a commonly used method to analyze immune reactivity and recellularization capacity. This strategy has many pitfalls that are not considered during the interpretation of results. The striking differences are in the healing response because rat skin and subcutaneous tissue have little in common with their human counterparts (Dorsett-Martin, 2004). In rats, loose-skinned healing occurs by wound contraction, which is the primary mechanism of wound healing instead of scarring followed by epithelization in humans. Additionally, rats can convert L-glucono-gamma-lactone to vitamin C within subcutaneous tissue, which acts as a powerful antioxidant and scar-prevention factor (Weber et al., 2019). As a result, implanted tested biomaterials appeared not to be susceptible to scarring, but in fact, they would trigger a different response in humans. Sprouting and elongation of a new vessel within the scaffold are dependent on a three-dimensional biomaterial architecture. Highly porous biomaterials provide a better surface to support the formation of a branched vascular network. In terms of ureter reconstruction, a scaffold’s ability to sustain angiogenesis should be considered the most decisive parameter, critical for a successful outcome. The ureter’s blood supply is primarily composed of a highly dichotomized vascular network that penetrates the ureter’s wall superficially. In this situation, the scaffold must have a microporous structure ready for the ingrowth of these small caliber vessels. In the case of ureter tissue-engineered-based reconstruction, the strategy of utilizing pre-implantation before repairing a ureteral defect was tested. Zhang et al. (2012) exploited pre-implantation to create a tubular scaffold from the fibrous capsule, which was formed in the peritoneal cavity. Liao et al. (2013) used omental pre-implantation as an *in vivo* bioreactor to increase neovascularization in the construct.

Although preimplantation is a tempting idea because it theoretically enables the creation of a functional vascular network. This method was only tested in animal settings.

Zhao et al. (2019) recently demonstrated the most sophisticated approach for ureteral reconstruction so far. They used VECM, which is naturally rich in VEGF, for rabbit ureter reconstruction. The tubular graft underwent three weeks of maturation within the omentum to develop a branched vascular network. At two months post-ureter reconstruction, the histological evaluation showed a layered structure of the ureter with a multilayered urothelium over the organized, smooth muscle tissue.

In the case of the ureter, local hypoxia associated with insufficient angiogenesis would be revealed after a shorter period when compared to the urethra and the bladder. A narrow ureteric lumen and a tendency to collapse due to higher constant higher abdominal pressure make tissue-engineered ureter particularly prone to scarring. It is one of the reasons why no clinical trial has been conducted yet.

## Conclusion

It is undeniable that reconstructive urology needs advanced therapeutic modalities utilizing recent biotechnological advances to improve current treatment effectiveness. Tissue engineering focusing on manipulation with cells and biomaterials ideally fits the modern reconstructive urology development pathway. Nevertheless, due to inefficient translational research, a misunderstanding of clinical needs, a tendency toward overly enthusiastic result interpretation, and sometimes a questioning attitude among clinicians toward tissue engineering applied outside the experimental field, this method is marginally used in urology. Despite decades of research, tissue engineering is still in the early stages of revolutionizing urology. Only a forward-looking approach to tissue engineering research and reliable study reports with repeatable outcomes will accelerate this process.

## Members of the Trauma and Reconstructive Urology Working Party of the European Association of Urology Young Academic Urologists

Jan Adamowicz, Enrique Fes Ascanio, Andrea Cocci, Mikołaj Frankiewicz, Campos Juanatey, Guglielmo Mantica, Clemens M. Rosenbaum, Wesley Verla, Malte W. Vetterlein, Marjan Waterloos

## Glossary

AM: Amniotic membrane
ASMA: Alpha-smooth muscle actin
ATPs: Advanced Therapy Products
BAM: Bladder acellular matrix
BMSCs: Bone marrow mesenchymal stem cells
BP: Glutaraldehyde
CBD: Collagen binding domain
CBER: Center for Biologics Evaluation and Research
CBMPs: Cell-based Medicinal Products
CDI: Carbodiimide
DRG: Dorsal root ganglion
ECM: Extracellular Matrix
EMA: European Medicines Agency
FDA: Food and Drug Administration
GAGs: Glycosaminoglycans
GDNF: Glial cell line-derived neurotrophic factor
GMP: Good Manufacturing Practice
GP: Genipin
hiPSCs: Pluripotent stem cells
IC: Interstitial Cystitis
iPSCs: Induced pluripotent stem cells
MHC: Myosin heavy chain
MSC: Mesenchymal stem cells
NGF: Nerve growth factor
NIH: National Institutes of Health
PGA: Poly(glycolic acid)
PLA: Polylactic acid
PLGA: Poly(lactic-co-glycolic acid)
PLLA: Spiral poly (l-lactic acid
SIS: Small intestinal submucosa
SM- 22a: Smooth muscle 22 alpha
SMC: Smooth muscle cells
SUI: Stress urinary incontinence
TEMPs: Tissue Engineered Medical Products
UCs: Urothelial cells
USC: Urine-derived stem cells
VECM: Vessel extracellular matrix
VEGF: Vascular endothelial growth factor
